# MicroRNA let-7 regulates the expression of ecdysteroid receptor (ECR) in *Hyalomma asiaticum* (Acari: Ixodidae) ticks

**DOI:** 10.1186/s13071-019-3488-6

**Published:** 2019-05-15

**Authors:** Feng Wu, Jin Luo, Ze Chen, Qiaoyun Ren, Ronghai Xiao, Wenge Liu, Jiawei Hao, Xiaocui Liu, Junhui Guo, Zhiqiang Qu, Zegong Wu, Hui Wang, Jianxun Luo, Hong Yin, Guangyuan Liu

**Affiliations:** 1 State Key Laboratory of Veterinary Etiological Biology, Key Laboratory of Veterinary Parasitology of Gansu Province, Lanzhou Veterinary Research Institute, Chinese Academy of Agricultural Science, Xujiaping 1, Lanzhou, 730046 Gansu People’s Republic of China; 2 Gansu Agricultural University, No. 1 Yingmen Village, Anning District, Lanzhou, 730070 Gansu People’s Republic of China; 3 Inspection and Comprehensive Technology Center of Ruili Entry-Exit Inspection and Quarantine Bureau No. 75, Ruihong Road, Ruili, 678600 Yunnan People’s Republic of China; 4 Jiangsu Co-innovation Center for Prevention and Control of Important Animal Infectious Diseases and Zoonoses, Yangzhou, 225009 People’s Republic of China; 5 Department of Engineering, Institute of Biomedical Engineering (IBME), University of Oxford, Oxford, OX3 7DQ UK

**Keywords:** *Hyalomma asiaticum*, microRNA let-7, Ecdysone receptor, 20-hydroxyecdysone, Molting regulation

## Abstract

**Background:**

Ticks are blood-sucking arthropods that can transmit diseases to humans and animals. These arthropods are the second most important vectors of pathogens. MicroRNAs are a class of conserved small noncoding RNAs that play regulatory roles in gene expression at the post-transcriptional level. Molting is an important biological process in arthropods. Research on the molting process is important for understanding tick physiology and control.

**Methods:**

Dual-luciferase reporter assays were used to assess the role of miRNA let-7 in ecdysteroid receptor (ECR) biology. The expression levels of ECR and let-7 were measured by real-time qPCR before and after tick molting. To explore the function of let-7 and ECR, we performed overexpression and knocking down of let-7 and RNAi of ECR in tick nymphs. The biological function of let-7 in molting was explored by injecting nymphs, ten days after engorgement, with let-7 agomir for overexpression and let-7 antagomir for knocking down. The rate of molting was then determined. ECR dsRNA was injected into ticks to evaluate the function of ECR by gene silencing. The expression of ECR and let-7 was measured using RT-qPCR. All data were analyzed using GraphPad Prism v.6.

**Results:**

The results of the luciferase assay using a eukaryotic expression system revealed that ECR was a natural target of let-7. Let-7 overexpressed by agomir affected the rate of molting (*P *< 0.01) and the period of molting (*P *< 0.01). Let-7 antagomir for knockdown affected the period of molting (*P *< 0.01), but there was no effect on the rate of molting (*P *= 0.27). ECR dsRNA gene silencing significantly affected the rate of molting (*P *< 0.05).

**Conclusions:**

This study demonstrated that let-7 can regulate the expression of ECR and that let-7 can affect molting in ticks. Our results help to understand the regulation of let-7 by 20-hydroxyecdysone (20E) and will provide a reference for functional analysis studies of microRNAs in ticks.

## Background

Ticks are blood-sucking arthropods that secrete immunomodulatory molecules to antagonize the inflammatory and immune responses of the host [1, 2]. Ticks are the second most important vectors of bacteria, viruses, parasites and other pathogens [3]. Medically important tick-borne diseases, including tick-borne encephalitis, granulocytic ehrlichiosis, babesiosis and Lyme disease, have a huge impact on human health [4, 5].

MicroRNAs (miRNAs) are a class of small non-coding RNAs with a length of 21–24 nucleotides. These molecules are involved in the post-transcriptional regulation of mRNAs in plants and animals [6, 7]. There are many miRNAs in multicellular organisms, and some are evolutionarily conserved [8]. Let-7 is the second miRNA identified in *Caenorhabditis elegans* and is evolutionally conserved in bilateral animals [6, 9]. This molecule is essential in the heterochrony pathway, playing a role in the regulation of developmental events [10]. In *Drosophila*, let-7 has been implicated in developmental timing [10, 11], wing development [12] and neurogenesis [13]. It also participates in innate immunity by targeting the antimicrobial peptide diptericin [14].

Let-7 is conserved in several species. The expression of four miRNAs, mir-34, mir-100, mir-125 and let-7, undergo temporal changes at larval-to-pupal or pupal-to-adult stages, and respond to ECD (steroid 20-hydroxyecdysone) and/or JH (juvenile hormone) in S2 cells of *Drosophila* [15]. In a study of silk worms [16], let-7 was shown to regulate molting and metamorphosis. Based on these reports, we hypothesized that let-7 could regulate molting and metamorphosis in *Hyalomma asiaticum*.

20-hydroxyecdysone (20E), a ubiquitous steroid hormone, coordinates multiple developmental events by recruiting the complex 20E-ECR/USP (ultraspiracle protein, USP), which directly activates the expression of many early-response genes, including E74, E75 and Broad-Complex. In turn, the products of early-response genes trigger the expression of late-response genes [17–19]. Ecdysone triggers molting and metamorphosis in insects. During metamorphosis, most cells are regulated by hormones, and cells from different tissues are regulated by different developmental pathways. In addition, ecdysone plays an essential role in oogenesis [20]. In a study of *Drosophila*, 20E-induced broad-complex could regulate miRNAs (let-7, miR-100, miR-34 and miR-125) which are involved in the 20E signaling pathway [21]. The present study shows that let-7 can regulate expression of ECR and molting in *H*. *Asiaticum* and provides an insight into the regulation of let-7 by 20-hydroxyecdysone (20E). The study may also serve as a reference for future research on the function of tick miRNA.

## Methods

### Tick collection

*Hyalomma asiaticum* ticks were collected from Gansu Province and have been maintained in our laboratory since 2006. Ticks were reared by feeding on rabbits for generations in the laboratory. All tick developmental stages were maintained at a temperature of 30 ± 2 °C and relative humidity of 80 ± 5% [22].

### Amplification of the 3′-UTR region of ECR

Total RNA was isolated from partially-engorged adult female ticks using Trizol reagent (TaKaRa, Shiga, Japan). First-strand cDNAs were synthesized using the protocol for transcriptase XL (Avian Myeloblastosis Virus, AMV) (TaKaRa) and oligo dT18. A 3′-RACE cDNA amplification kit (Invitrogen, California, USA) was used to obtain the 3′-sequences of the ECR genes of *H. asiaticum.* The gene-specific primers GSP-ECR-3UTR-F1 and GSP-ECR-3UTR-F2 were used in 3′-RACE. Nested PCR was performed to amplify the sequence of 3′-UTR. The first PCR was performed using primers GSP-ECR-3UTR-F1 and AUAP (universal amplification primer) by denaturing at 95 °C for 3 min followed by 35 cycles at 95 °C for 30 s, 55 °C for 30 s and 72 °C for 90 s, and a final extension step at 72 °C for 10 min. Nested PCR was performed using the first-round PCR product (diluted 100 times in RNase-free water) as a template and primers AUAP and GSP-ECR-3UTR-F2. The PCR product was purified and cloned into the PMD 19-T vector (TaKaRa). Gene sequencing was performed by GenScript (Nanjing, China).

### Cloning of let-7

The primers and loop primer were designed according to the mature sequence of isc-let-7 (MIMAT0012680, miRBase). The loop primer (5′-GTC GTA TCC AGT GCA GGG TCC GAG GTA TTC GCA CTG GAT ACG ACA CTA TAC A-3′) was used to synthesize the first-strand cDNA. PCR was performed using let-7-F, let-7-R and the cDNA by denaturing at 95 °C for 3 min, followed by 35 cycles at 95 °C for 30 s and 60 °C for 30 s, and a final extension step at 60 °C for 5 min. PCR was performed to amplify the sequence; the PCR product was ligated into a PMD 19-T vector (TaKaRa), cloned into JM109 *E. coli* (Takara) and sequenced by GenScript (Nanjing, China).

### Prediction of target sites

The mature let-7 miRNA sequences (UGAGGUAGUAGGUUGUAUAGU) were obtained from miRBase release 19. Two additional ECR 3′-UTR sequences of *Amblyomma americanum*, AamECR3′-UTR-1 (AF020189.1) and AamECR3′-UTR-2(AF020190.1), along with *H. asiaticum* ECR 3′-UTR were obtained from NCBI to predict the target sites. The miRNA target binding sites in the 3′-UTR of ECR were predicted using the miRNA prediction software RNAhybrid [23, 24].

### Dual luciferase reporter (DLR) assay

let-7 mimic and negative control (NC) were synthesized by RiboBio (Guangzhou, China). miRNA mimics are small, chemically modified double-stranded RNAs that mimic endogenous miRNAs and enable miRNA functional analysis by upregulation of miRNA activity. The miRNA negative control is a mimic and the sequence was based on a *Caenorhabditis elegans* miRNA, which was not similar to mammalian or tick miRNAs. Mammalian BHK-CDC cell lines were used in the DLR assay to ensure high transfection efficiency and low background expression of let-7. ECR-A2 wild-type (WT) or mutant 3′-UTR was cloned into a pmirGLO vector (Promega, Madison, USA). BHK cells were transfected with 50 nM miRNA mimic (final concentration) and 0.8 ng pmirGLO reporter plasmid and mixed with 1 μl of Lipofectamine® 2000 Transfection Reagent® (Invitrogen, California, USA) and 50 μl of Opti-MEM Reduced Serum Medium (Gibco, California, USA) in each well of a 24-well plate. In this assay, let-7 effectively regulates the expression of the empty pmirGLO vector. Therefore, a 500-bp fragment not containing the ECR sites to which let-7 binds was cloned into the pmirGLO vector and this recombinant plasmid was designated as RP-NC. Similarly, the 3′-UTR of ECR was cloned into GLO plasmid named WT (3′-UTR-GLO). There were four groups in this experiment: RP-NC with miR-NC, WT with miR-NC, RP-NC with mimic, and WT with mimic. The group of RP-NC with miR-NC was used as a blank control, and these groups of RP-NC with mimic and WT with mimic were used as the negative controls. The Dual-Luciferase® reporter assay (Promega) was performed according to the manufacturer’s protocol 48 h after transfection. The assays were performed in triplicate for three times.

### Construction and transfection of plasmids

For the construction of plasmids, a 1358-bp ECR sequence was used, including an ORF (960-bp) and 3′-UTR (398-bp), PCR was performed using cDNA and performed under the following conditions: 95 °C for 3 min followed by 35 cycles at 95 °C for 30 s, 55 °C for 30 s and 72 °C for 30 s, and a final step at 72 °C for 10 min. The sequence was cloned into PcDNA3.1^(+)^ plasmid (Invitrogen) using restriction sites *EcoR*I and *Not*I. The resulting plasmid was expressed in BHK-CDC cells. The PCR primers are listed in Table 1.

**Table 1:** Primers for this experiment

Name	Sequence (5ʹ–3ʹ)
ECR-DP	F:CTGYGACATCGACATGTACATG
	R:TCACTCYTGGATGTCCCARATCTC
dsECR	F:GGATCCTAATACGACTCACTATAGGCATCGTCTATGGGTGGTGGTGTGAG
	R:GGATCCTAATACGACTCACTATAGGCGATGGCTGTGAGGAGTGCATATTC
dsNC-1	F:GTAGCAGGTGTGGTTCATCC
	R:CTGATGCATTGCCTTCGTCC
dsNC	F:GGATCCTAATACGACTCACTATAGGGTAGCAGGTGTGGTTCATCC
	R:GGATCCTAATACGACTCACTATAGGCTGATGCATTGCCTTCGTCC
ECR-WT-DLR	F:AGCTTTGTTTAAACGCCGAGATTTGGGACATCCAAGAG
	R:CTAGTCTAGACAGAAAAGAGGGTTAGATTCGC
RP-NC-DLR	F:AGCTTTGTTTAAACGCCGAGATTTGGGACATCCAAGAG
	R:CTAGTCTAGACAGAAAAGAGGGTTAGATTCGC
ECR-qPCR	F:GTGCCAGTGTAGGCGATTCAG
	R:CTGTATGCGCTCCACCTTGC
GSP-ECR-3UTR-F1	F:TGGACCCGTGCAAGGTGGAG
GSP-ECR-3UTR-F2	F:ACAGAGCTGCGCACCTTGG
β-actin	F:CGTTCCTGGGTATGGAATCG
	R:TCCACGTCGCACTTCATGAT
GAPDH	F:CGTGCCGCCTGGAGAAACCTG
	R:AGAGTGGGAGTTGCTGTTGAAGTCG
let-7-qPCR	F:ACACTCCAGCTGGTGAGGTAGTAGGT
miR-2a-qPCR	F:ACACTCCAGCTGGTATCACAGCCAGCTT
miR-8-qPCR	F:ACACTCCAGCTGGTAATACTGTCAGGTA
mir-451-qPCR	F:ACACTCCAGCTGGAAACCGTTACCATTA
miRNA-URP	R:GTCGTATCCAGTGCAGGGTCCGAGGT
ECR-NOTI-1429	F:CCGGAATTCATGCGACGCAAGTGCCA
	R:AAATATGCGGCCGCTGCATGAGCAGAAAAGAGGG

*Note*: R= A/G, W= A/T, Y= T/C

The cells were seeded at 10^6^ cells per well in 24-well plates 12 h before transfection. The cells were transfected with 0.8 µg of plasmid and 50 nM miRNA mimics (RIBOBIO, Guangzhou, China) per well. No-mimic treatment cells were used in the blank control. The cells were transfected with WT plasmid and mimic in the negative controls. The cells were lysed after 48 h in 1 ml of Trizol. Finally, real-time PCR was performed using rat GAPDH as the reference gene.

### Real-time quantitative PCR

The expression levels of let-7 and ECR in *H. asiaticum* were estimated by RT-qPCR. Total RNA from different developmental stages of ticks was extracted using Trizol reagent, and the first strand cDNAs were synthesized from total RNA using stem-loop primers for let-7 or oligo dT(18) for the ECR gene. Subsequently, 1 μg of total RNA was reverse transcribed using stem-loop primers (SL-primers) for generating miRNA cDNAs using PrimeScript™ RT reagent kit with gDNA Eraser (TaKaRa). The miRNA stem-loop primer and miRNA RT-qPCR primers were designed as previously described [25]. The primer sequences used in this study are listed in Table 1.

ECR primers were designed to measure ECR expression by qPCR in Table 1. RT-qPCR for miRNA or mRNA was performed using the SYBR® Premix *Ex Taq™ II* Kit (TaKaRa) and an MX7500 detection system (Siskiyou, Inc., Grants Pass, OR, USA).

### Injection of miRNA

To further validate the role of let-7, miRNA was overexpressed using let-7 agomir *in vivo*. agomir is a chemically-modified double-strand miRNA mimic. Its antisense strand is modified: 2 phosphorothioates at the 5′ end, 4 phosphorothioates at the 3′ end, 3′ end cholesterol group and full length nucleotide 2′-methoxy modification. The developmental period from fully-engorged larvae to unfed nymphs was 7–10 days and from fully-engorged nymphs to unfed adults was 30–45 days. The nymphs received the miRNAs 10 days after blood-feeding and were reared in the laboratory.

The miRNA or miRNA negative control (miRNA-NC) (RiboBio) was microinjected into the haemocoel of engorged nymphs at a dose of 400 nm in 0.5 μl [26]. Each miRNA was injected into 100 nymphs 10 days after blood-feeding. Control ticks were injected with an equal volume of PBS buffer (pH 7.4). In this experiment, the ticks were divided into four groups as follows: non-injection (untreated), PBS-injection (PBS), NC-miRNA-injection (miR-NC) and let-7-agomir-injection (agomir). Twenty-four hours after injection, dead ticks were removed and live ticks were maintained at a temperature of 25 °C and relative humidity of 75–85%. The total RNA from three live ticks was extracted for RT-qPCR. Three miRNAs (miR-2a, miR-8 and miR-451) were tested by RT-qPCR. The miR-2a loop primer (5′-GTC GTA TCC AGT GCA GGG TCC GAG GTA TTC GCA CTG GAT ACG AC GCT CAT CA-3′), the miR-8 loop primer (5′-GTC GTA TCC AGT GCA GGG TCC GAG GTA TTC GCA CTG GAT ACG AC GAC ATC TT-3′) and the miR-451 loop primer (5′-GTC GTA TCC AGT GCA GGG TCC GAG GTA TTC GCA CTG GA TAC GAC AAA CTC AG-3′) for miR-2a, miR-8 and miR-451 first-strand cDNA, respectively. Ticks were observed on a daily basis and survived for 1 month. Dead ticks were removed and the survival rates were calculated after 1 month.

Antagomirs also known as anti-miRs or blockmirs are a class of chemically engineered oligonucleotides that prevent other molecules from binding to a desired site on an mRNA molecule. Antagomirs are used to silence endogenous microRNA (miR) [27]. The miRNA antagomir or miRNA negative control (miRNA-NC) (RiboBio) was micro-injected into the haemocoel of engorged nymphs at a dose of 400 nm in 0.5 μl. Each miRNA was injected into 100 nymphs 10 days after blood-feeding. Control ticks were injected with an equal volume of PBS buffer (pH 7.4). In this experiment, the ticks were divided into four groups as follows: non-injection (untreated), PBS-injection (PBS), NC-miRNA-injection (miR-NC) and let-7-antagomir-injection (ant-let-7). Twenty-four hours after injection, dead ticks were removed and live ticks were maintained at a temperature of 25 °C and relative humidity of 75–85%.

### RNA interference

A 572-bp fragment corresponding to the core region of ECR and a 539-bp NC fragment were amplified by PCR. NC is a plant-derived gene. The primers used were ECR-RNAi-F, ECR-RNAi-R for synthetic dsRNA of ECR named dsECR, and NC-RNAi-F, NC-RNAi-R for dsRNA of NC named dsNC. PCR was performed under the following conditions: 95 °C for 3 min followed by 35 cycles at 95 °C for 30 s, 55 °C for 30 s and 72 °C for 30 s, with a final step at 72 °C for 10 min. The dsRNA was prepared using the T7 RiboMax™ express RNAi system (Promega) according to the manufacturer’s instructions. In this experiment, the ticks were divided into four groups as follows: non-injection (untreated), PBS-injection (PBS), dsNC-injection (dsNC) and dsECR-injection (dsECR). Synthetic dsRNAs were microinjected into 100 nymphs. The nymphs received the dsRNA 10 days after blood-feeding and were reared in the laboratory. A total of 2 μg of dsRNA in 0.5 μl of water was injected into each tick and the negative control groups included a PBS-injection group (untreated), a group receiving PBS (PBS group), and a group receiving NC dsRNA (ds-NC group). Subsequently, the expression of ECR genes was measured by quantitative real-time PCR. Statistical analysis was conducted to assess the effect of RNA silencing on molting.

### Hormone treatment

Ten days after blood-feeding, the nymphs were injected with either 3 ug of 20E dissolved in 0.5 ul of water (experimental group) or water of DMSO (control group). let-7 and ECR were tested by qPCR at 24 and 48 h. β-actin was amplified as internal controls. The relative expression of miRNAs and ECR was calculated by the 2^−ΔΔCt^ method.

### Statistical analysis

All data sets are shown as means ± SEM (*n* ≥ 3). The dual luciferase reporter (DLR) assay and quantitative real-time PCR results were analyzed using a two-tailed unpaired Student’s t-test as detailed in the figure legends by GraphPad Prism 6 software (GraphPad Software, San Diego, CA, USA). Significance was set at *P *< 0.05.

## Results

### Analysis of target genes and let-7 binding sites

**Fig. 1:**
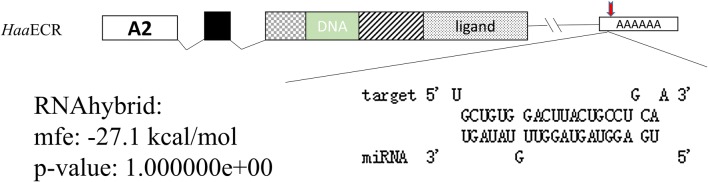
Predicted let-7 target sites in the ECR gene by RNAhybrid. The red triangle indicates the binding sites for let-7 in 3’-UTR. *Haa*EcR was identified to predict the binding site with let-7 in this experiment

The partial sequence of ECR include a 960-bp opening reading frame (ORF) and a 398-bp 3′-UTR containing a poly (A) terminal sequence (*Haa*ECR, GenBank: MF135614.2). Additionally, we identified three *Aam*ECR isoforms (*Aam*ECRA1, *Aam*ECRA2 and *Aam*ECRA3) and the *Aam*ECR3′-UTR isoforms in *H. asiaticum*. The structure of the gene encoding *Haa*ECR is shown in Fig. 1, redrawn from the study by Guo et al. [28]. *Aam*ECR and *Haa*ECR were used for predicting the let-7 binding site and these sequences contained the same let-7 binding site (Fig. 1).

### MiRNA represses ECR expression in BHK-CDC cells

**Fig. 2:**
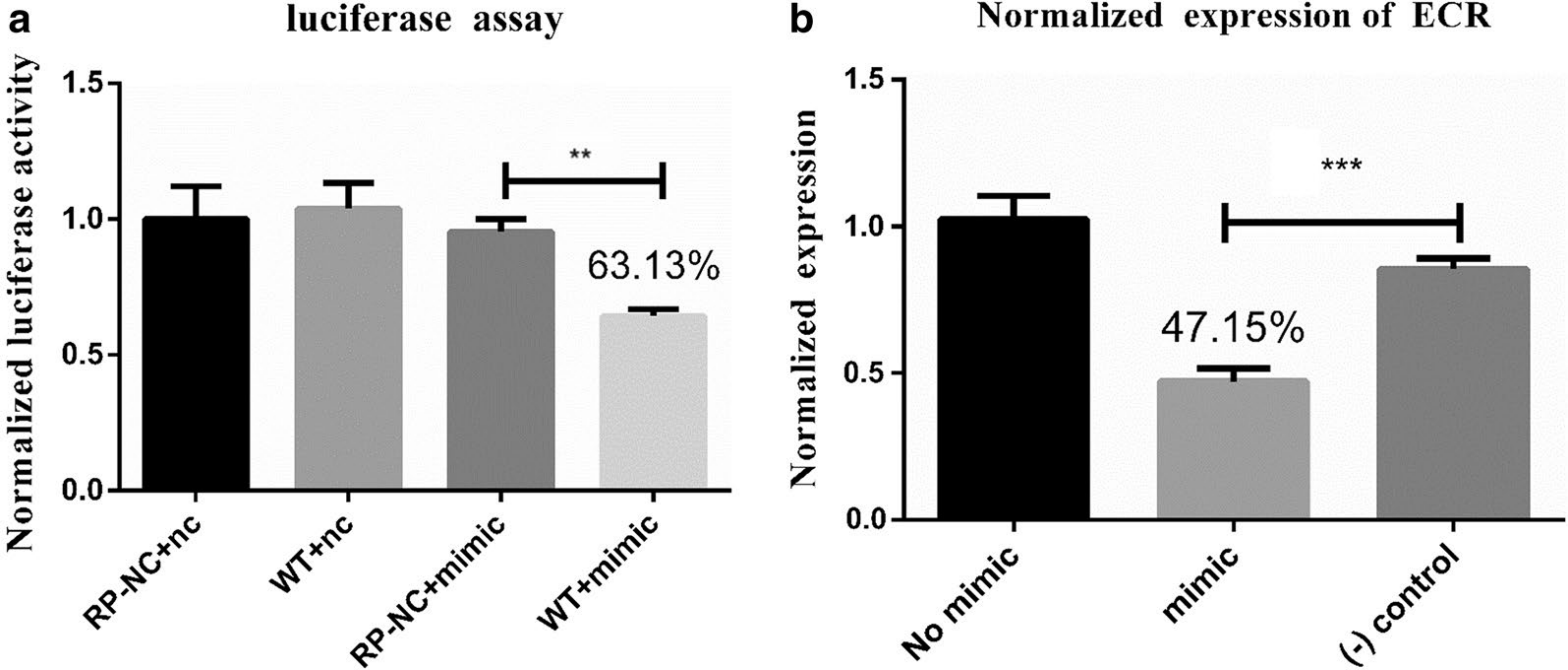
**a** ECR is a target of let-7. Dual luciferase reporter assay results are represented as mean ± SEM of triplicate samples. **b** The recombinant plasmid pcDNA3.1 containing the ECR gene, including a 960-bp ORF and a 298-bp 3-UTR, was transfected into BHK cells. The expression of ECR was measured by qPCR. Data are presented as the mean ± SEM of triplicate samples. *P < 0.05, **P < 0.01, ***P < 0.001 (Student’s t-test)

The DLR assay was used to assess the effect of let-7 on ECR expression in BHK-CDC cells. The plasmids with mimic or NC sequences were transfected into BHK-CDC cells. Compared with the RP-NC+NC group, the ratio of luciferase activity was 63% (Fig. 2a). The results showed that the ratio of luciferase activity in the WT+mimic group was different from that in the other groups.

To determine whether or not let-7 could repress ECR expression, the eukaryotic expression vector containing the 1358-bp ECR gene, including a 960-bp CDS and a 398-bp 3′-UTR, was transfected with the let-7 mimic into BHK-CDC cells (Fig. 2b). ECR expression in the treatment group was 47% when compared with the control group. These results demonstrated that ECR was the target of let-7 and that let-7 repressed ECR expression in BHK cells.

### Analysis of the expression of let-7 and ECR

**Fig. 3:**
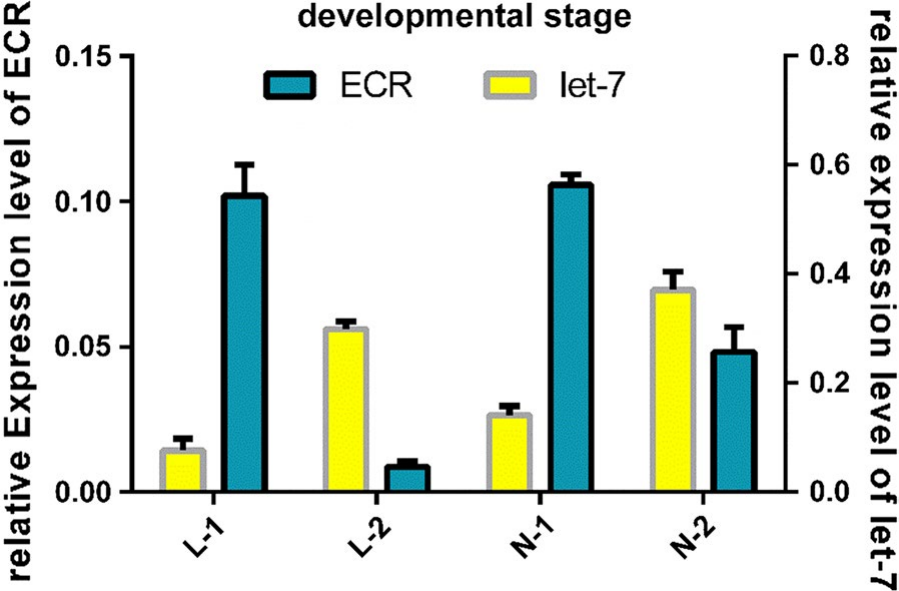
Analysis of the expression of let-7 and ECR. L-1 and L-2 correspond to engorged larvae before and after molting, respectively. N-1 and N-2 correspond to engorged nymphs before and after molting, respectively. Data are presented as the mean ± SEM of triplicate samples

Total RNA was extracted from different stages of *H. asiaticum* and the expression levels of let-7 and ECR were amplified by RT-qPCR at two molting stages: from larvae to nymphs and from nymphs to adults. β-actin was amplified as internal controls. Relative expression of miRNAs and ECR was calculated by the 2^−ΔCt^ method. In Fig. 3, L-1 and L-2 correspond to engorged larvae before and after molting, respectively, and N-1 and N-2 correspond to engorged nymphs before and after molting, respectively. For both engorged larvae and engorged nymphs, the levels of ECR expression before molting were higher than those after molting (*t*_(4)_ = 6.920, *P * = 0.0023; *t*_(6)_ = 8.520, *P * < 0.001), whereas the expression levels of let-7 before molting were lower than after molting (*t*_(6)_ = 8.520, *P * < 0.001; *t*_(6)_ = 3.860, *P * < 0.0084). The differences were significant. Moreover, a correlation in expression levels between let-7 and ECR and a different changing tendency of the expression level were observed. Therefore, this analysis suggests that there is an interaction between let-7 and ECR, and this interaction affects molting. Such a difference was not due to the digestion of the tick blood meal before and after molting because the levels of let-7 and *Haa*ECR were low in the host blood.

### Overexpression of let-7 in *H. asiaticum*

**Fig. 4:**
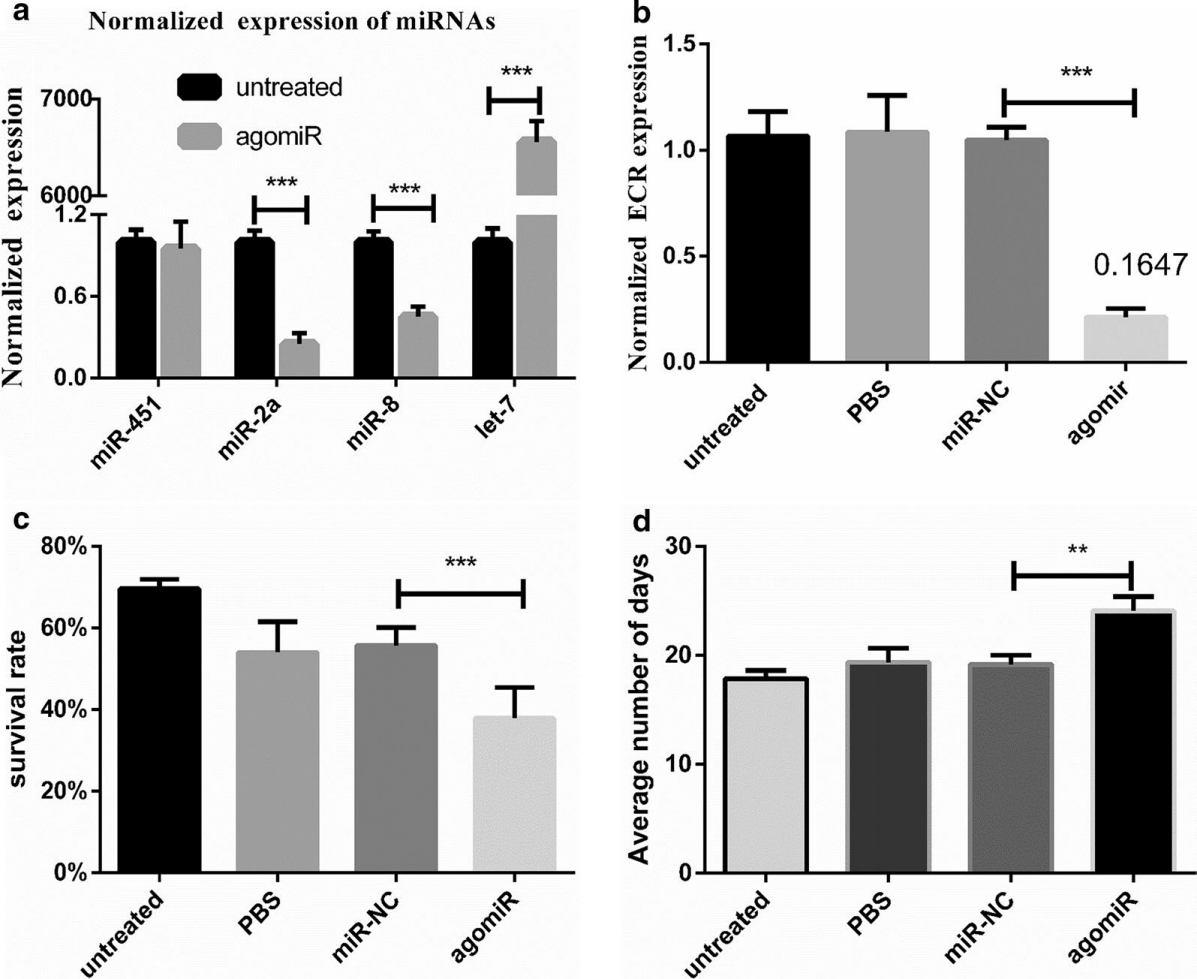
**a** The miRNAs were tested in the agomir and untreated groups. **b** The expression of ECR was tested 24 h after injection of agomir. **c** Effect of let-7 overexpression on survival rate. **d** Average number of days of molting after injection with agomir. Data are presented as the mean ± SEM of triplicate samples. *P  < 0.05, **P  < 0.01, ***P  < 0.001

let-7 agomir was used to elucidate the biological function of let-7 in ticks. After 24 h of miRNA injection, the expression of let-7 in the agomir group was increased approximately 6935-fold and the expression of ECR was decreased approximately 6.04-fold compared to the miR-NC group. Three miRNAs (miR-2a, miR-8 and miR-451) were tested by RT-qPCR, and the expression levels of mir-2a and mir-8 were downregulated by approximately 74 and 52%, respectively, in the agomir group compared to the untreated group, whereas the expression of miR-451 was not significantly changed (Fig. 4a). There were significant differences in the expression levels of ECR between the agomir group and the other groups (*t*_(6)_ = 15.56, *P * < 0.001) (Fig. 4b). The tick survival rate in the let-7 overexpression group was approximately 30% less in comparison to the miR-NC group. However, the survival rate in the untreated group was greater than those in other groups (Fig. 4c). There were significant differences between the agomir group and the other groups (*t*_(4)_ = 3.514, *P *< 0.0024), showing that injection caused physical injury to ticks.

Moreover, our results showed that the period of molting was also affected by agomir treatment. In engorged ticks, the average number of days of molting was 18.20 ± 1.02 days in the untreated group, 19.37 ± 1.05 days in the PBS group, 19.16 ± 0.71 days in the miR-NC group, and 24.43 ± 0.62 days in the agomir group. The periods of molting in the PBS and miR-NC groups were not significantly different from that in the untreated group (*P *> 0.05), indicating that the period from engorgement to molting was not affected by the injection operation nor non-specific miRNA injection (Fig. 4d). However, molting was significantly delayed by the treatment of agomir (*t*_(4)_ = 5.874, *P * = 0.0042), demonstrating that let-7 overexpression prolonged the molting period in ticks.

**Fig. 5:**
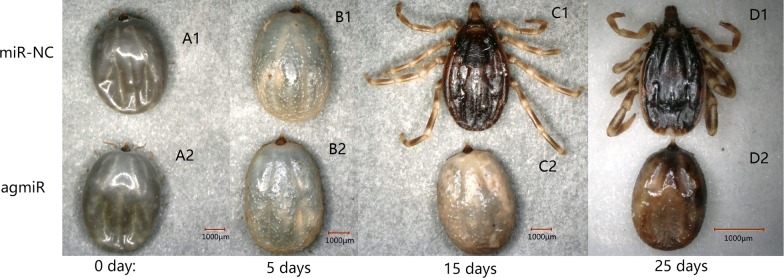
Tick morphology at 0, 5, 15 and 25 days after injection of let-7 agomir. Day 0 is defined as when nymphs received the miRNAs, 10 days after blood-feeding; they were reared in the laboratory. There was no difference between the agomir group (A2) and the control group (A1). On day 5, the phenotype had a slight difference in cuticular hardness. On day 15, most ticks of the control group had been through their cuticle, but most of the experiment group had not. The ticks’ cuticle of experiment (C2) was harder than control (C1). On day 25, there were no signs of life for ticks who had failed to be through their cuticle (D2). The cuticular hardness of ticks who had been injected with agomir of let-7 (D2) were much harder than ticks who had been injected with miR-NC (D1). *Scale-bars*: 1000 µm

Tick morphology was evaluated at 0, 5, 15 and 25 days post-injection (pi) of let-7 agomir or miR-NC (Fig. 5). Tick phenotype did not change until day 5 pi but presented significant difference after 5 days pi. Ticks from the agomir group exhibited a conspicuous “molting defect” phenotype, in which the old cuticle was not shed. These results demonstrate that the overexpression of let-7 could reduce the survival rate and increase the molting period. The results are shown in Table 2.

**Table 2:** The phenotype of ticks in the overexpression experiment

	Group			
	Untreated	PBS	miR-NC	agomir
Average number of days	18.20 ± 1.02^a^	19.37 ± 1.05^a^	19.16 ± 0.71^a^	24.43 ± 0.62^b^
Survival rate (%)	67.385 ± 1.085^a^	54.914 ± 0.184^b^	56.138 ± 0.736^b^	38.469 ± 1.182^c^

*Notes*: Each data point is the mean ± SEM of three independent experiments (*n *= 3). Different letters indicate significant differences on the same line (*P *< 0.05)

### ECR interference in *H. asiaticum*

**Fig. 6:**
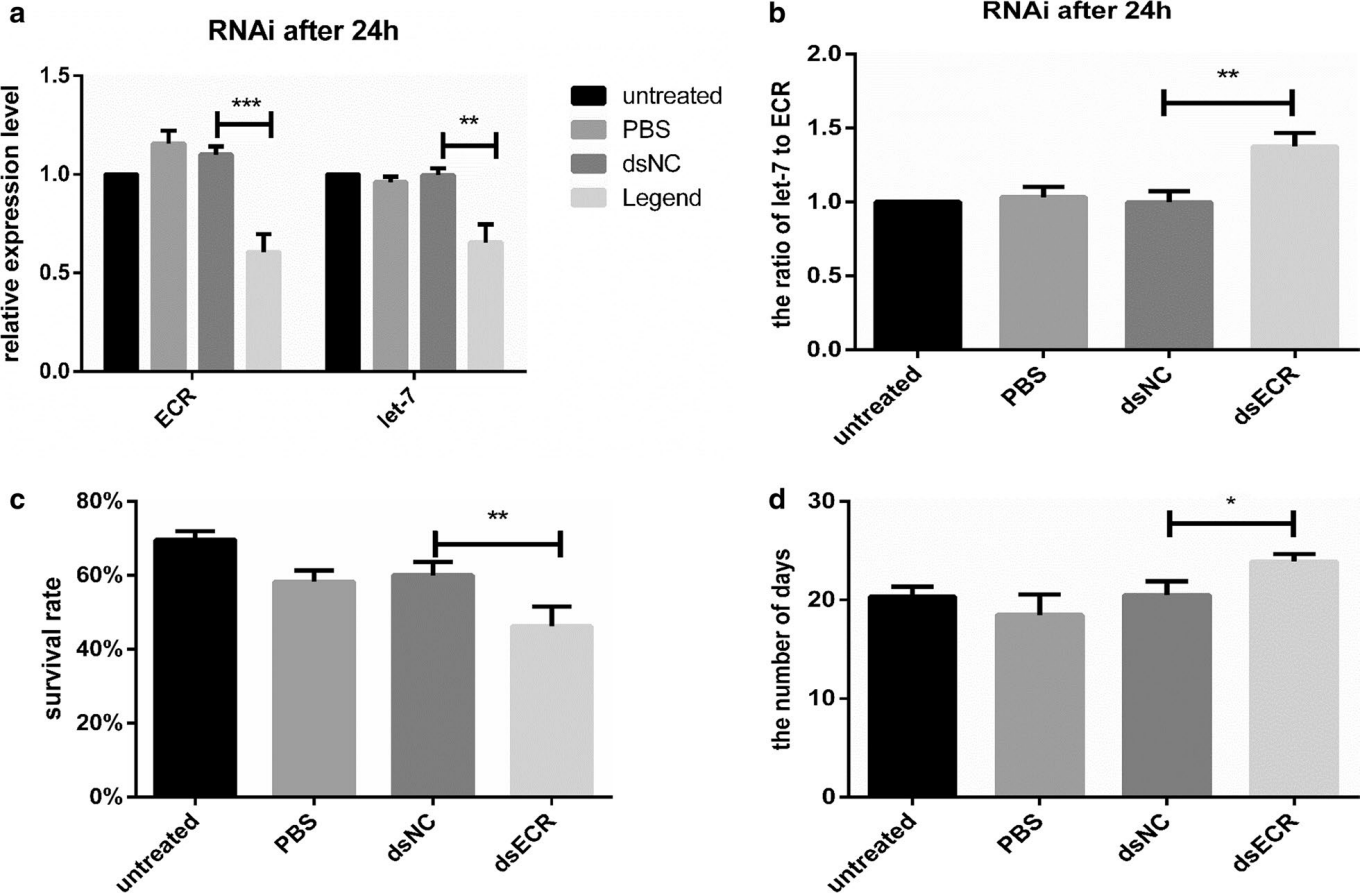
**a** ECR expression was determined 24 h after injection of dsRNA. **b** The ratio of let-7 to ECR 24 h after injection of dsRNA. Effect of RNA interference on survival rate. **d** Average number of days of molting after injection of dsRNA. Data are presented as the mean ± SEM of triplicate samples. *P  < 0.05, **P  < 0.01, ***P  < 0.001

The biological function of ECR was confirmed *in vivo* by injecting dsECR in engorged nymphs to silence the ECR gene. After the treatment, the expression levels of ECR and let-7 were measured by real-time PCR 24 h post-injection. ECR expression in the dsECR group was decreased approximately by 42% and let-7 was upregulated approximately by 37% compared to the untreated group (Fig. 6a). The ratio of let-7 to ECR was increased (*t*_(4)_ = 5.492, *P * = 0.017) approximately 1.37 times in the dsECR group compared with the untreated group (Fig. 6b). The effect of RNA interference on the survival rate is shown in Fig. 6c. The survival rate in the dsECR group was significantly decreased by approximately 8% compared with the dsNC group (*t*_(4)_ = 5.192, *P * = 0.0067). In engorged ticks, the average number of days of molting was 20.34 ± 1.46 days in the untreated group, 18.48 ± 2.08 days in the PBS group, 21.49 ± 0.42 days in the miR-NC group and 23.92 ± 0.74 days in the agomir group (Fig. 6d). The results are shown in Table 3.

**Table 3:** The phenotype of ticks in the RNAi experiment

	Group			
	Untreated	PBS	dsNC	dsECR
Average number of days	20.34 ± 1.46^a^	18.48 ± 2.08^a^	21.49 ± 0.42^a^	23.92 ± 0.74^b^
Survival rate (%)	62.468 ± 3.146^a^	50.451 ± 0.791^a^	54.264 ± 1.304^a^	45.614 ± 3.079^b^

*Notes*: Each data point is the mean±SEM of three independent experiments (*n *= 3). Different letters indicate significant differences on the same line (*P *< 0.05)

### Let-7 silence by antagomir

**Fig. 7:**
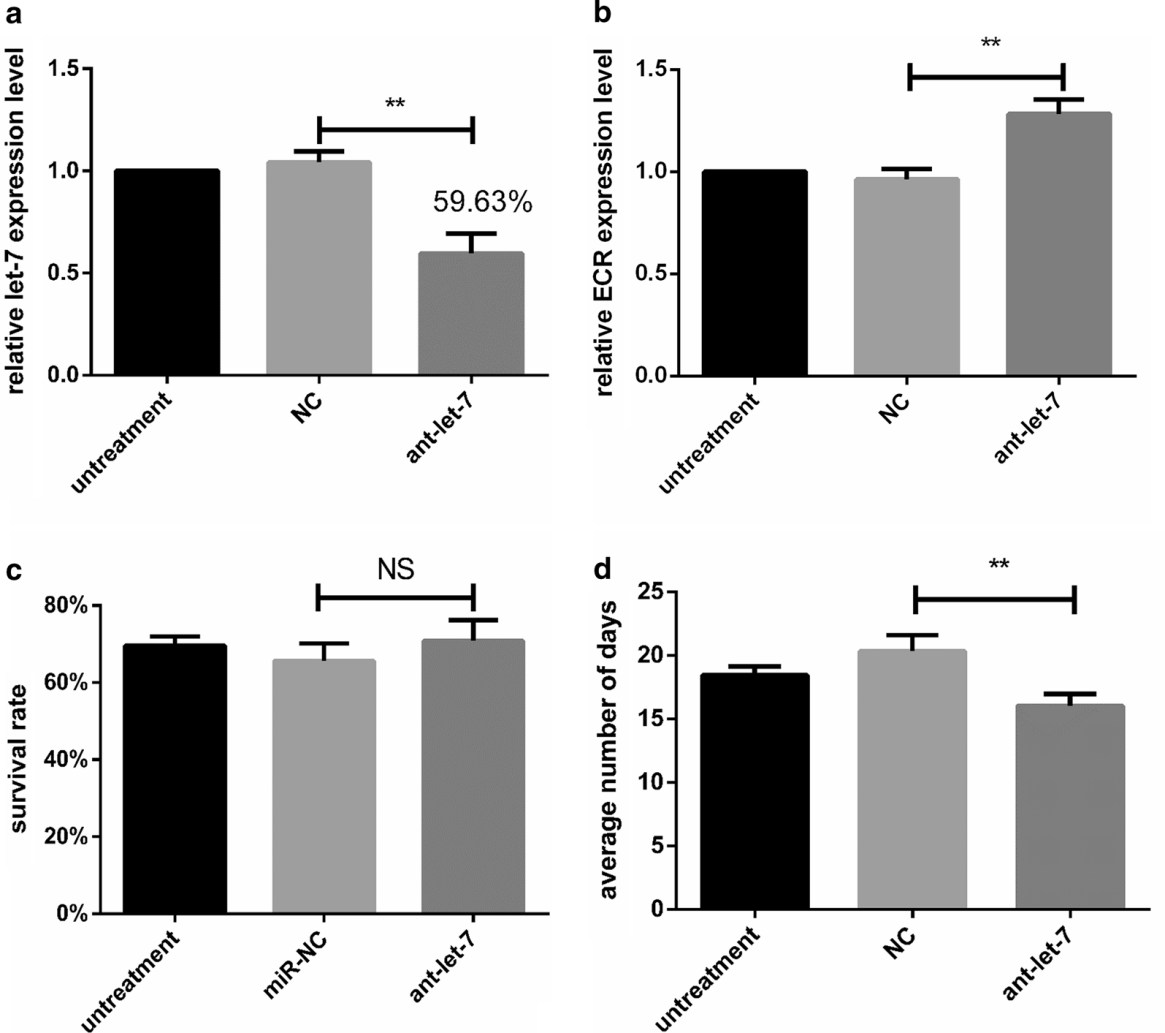
**a** ECR expression was determined 24 h after injection of antagomir. **b** Let-7 expression was evaluated 24 h after injection of antagomir. **c** Effect of miRNA interference on survival rate. **d** Average number of days of molting after injection of antagomir. Data are presented as the mean ± SEM of triplicate samples. *P  < 0.05, **P  < 0.01, NS, not significant

To explore the effect of let-7 silencing in ticks, a let-7 antagomir (ant-let-7) was used in this experiment to knock down the expression of let-7. The missense sequence of let-7 was used as the negative control of the antagomir. Compared with NC, the let-7 expression of antagomir injection group was knocked down approximately to 59% (*t*_(4)_= 6.961, *P * = 0.022) (Fig. 7a) at 24 h and *haa*ECR increased 1.26 times (*t*_(4)_ = 4.908, *P *= 0.0080) (Fig. 7b). The effect of let-7 interference on the survival rate is shown in Fig. 7c. The survival rate in the ant-let-7 group was not significantly different from the NC group (*t*_(4)_ = 1.278, *P * = 0.2702). Moreover, our results showed that the period of molting was also affected by antagomir treatment. In engorged ticks, the average number of days of molting was 18.44 ± 0.4075 (*n *= 3) days in the untreated group, 20.32 ± 0.7421 (*n * = 3) in the miR-NC group and 16.04 ± 0.5410 (*n * = 3) days in the antagomir group (Fig. 7d). The results are shown in Table 4.

**Table 4:** The phenotype of ticks in the let-7 disturbed experiment

	Group		
	Untreated	miR-NC	ant-17
Average number of days	18.44 ± 0.4075^a^	20.32 ± 0.7421^a^	16.04 ± 0.5410^b^
Survival rate (%)	69.672 ± 1.335^a^	65.487 ± 2.603^a^	70.864 ± 3.074^a^

*Notes*: Each data point is the mean ± SEM of three independent experiments (*n *= 3). Different letters indicate significant differences on the same line (*P *< 0.05)

## 20E influences expression of let-7 and ECR

**Fig. 8:**
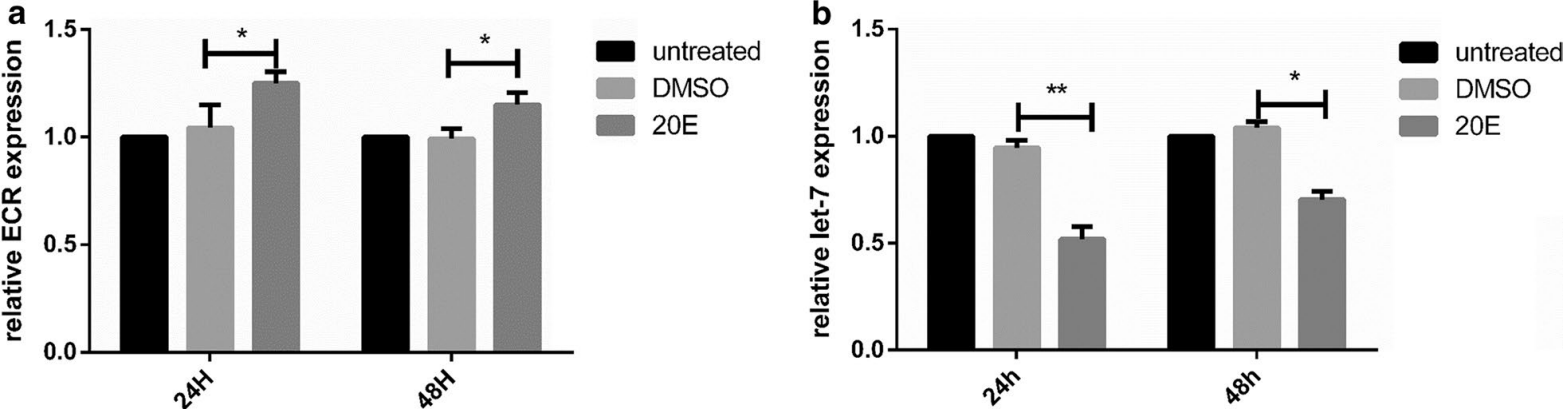
**a** ECR expression was determined 24 h after injection of 20E. **b** Let-7 expression was evaluated 24 h after injection of 20E. Data are presented as the mean ± SEM of triplicate samples. *P  < 0.05, **P  < 0.01

Compared with the DMSO-injected group, expression of let-7 was downregulated by approximately 48 and 30% at 24 and 48 h, respectively; ECR was upregulated by approximately 21 and 16% at 24 and 48 h, respectively (Fig. 8a, b).

### These miRNAs were analyzed by 3′-UTR of ECR

**Fig. 9:**
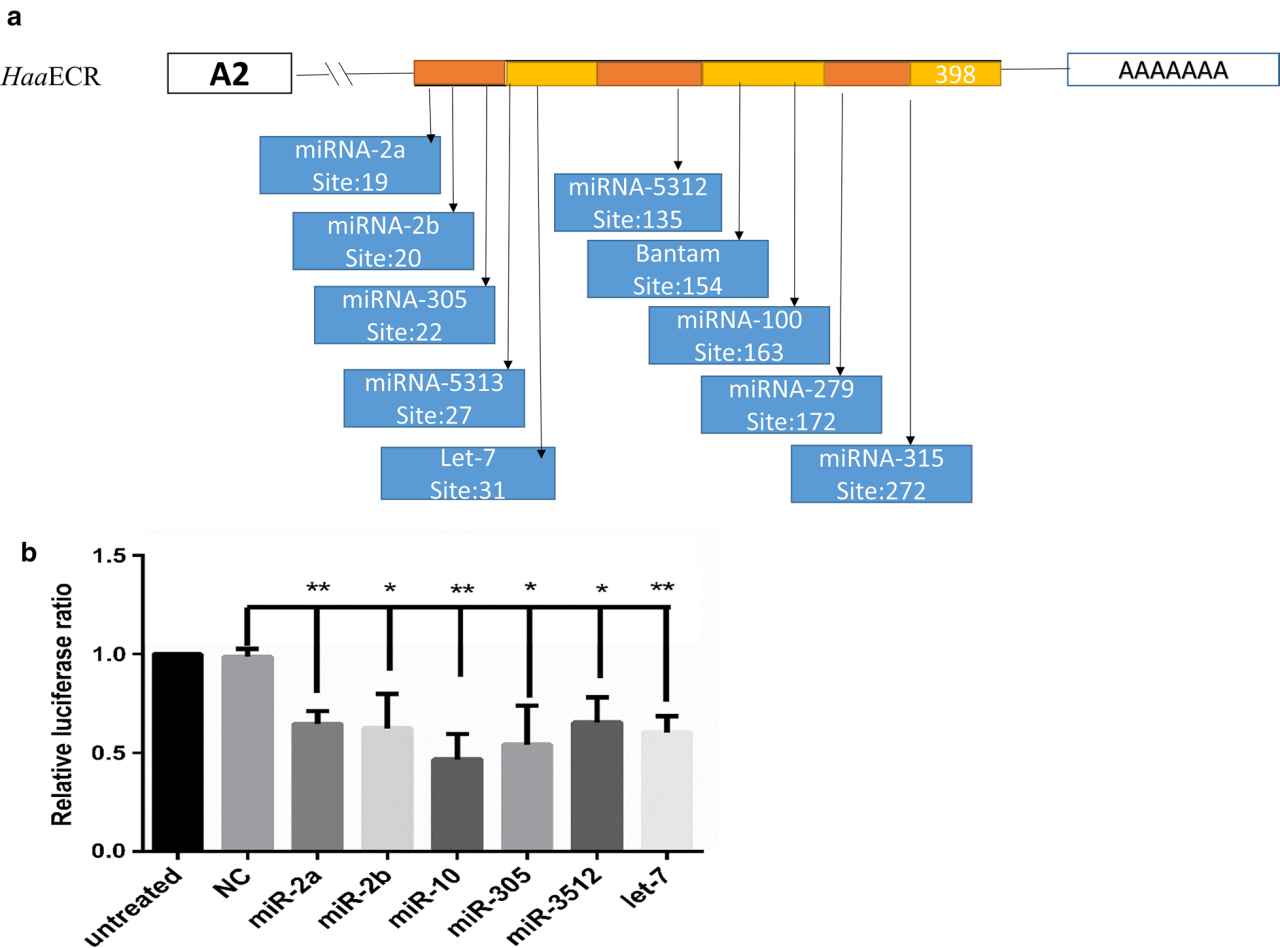
**a** Predicted target sites in the 3’-UTR of ECR gene and miRNAs by RNAhybrid. **b** Dual luciferase reporter assay results showed five miRNAs (miR-2a, miR-2b, miR-10, miR-305 and miR-5312) were able to regulate ECR. Note that this is only a suggestion based on the available data. Data are presented as the mean ± SEM of triplicate samples. *P  < 0.05, **P  < 0.01

All miRNAs from *Ixodes scapularis* in miRBase web and 3′-UTR of ECR were analyzed by RNAhybrid software (Fig. 9a). Then, the expression of these miRNAs was tested by qPCR and it was found that five miRNAs (miR-2a, miR-2b, miR-10, miR-305 and miR-5312) were upregulated when let-7 was silenced; these were downregulated when let-7 was overexpressed (Table 5). RNAhybrid software Luciferase assays showed that these five miRNAs (miR-2a, miR-2b, miR-10, miR-305 and miR-5312) can regulate the expression of ECR *in vivo* (Fig. 9b).

**Table 5:** miRNA relative expression level

	Agomir	Antagomir
let-7	Up	Down
miR-2a	Down	Up
miR-2b	Down	Up
miR-10	Down	Up
miR-305	Down	Up
miR-5313	Up	Up
miR-5312	Down	Up
miR-279	Down	Down
miR-100	Up	Up
bantam	Down	Down
miR-315	Down	ns

*Notes*: miRNAs were tested when let-7 is overexpressed and knocked down. Compared with the group of injected miRNA-NC, “down” means that the expression of miRNAs is downregulated, “up” means that the expression of miRNAs is upregulated and “ns” means not significant

## Discussion

The steroid hormone 20E triggers significant developmental changes in *Drosophila*, including molting and metamorphosis, and provides a model system for defining the developmental and molecular mechanisms of steroid signaling. 20E acts *via* a heterodimer of two nuclear receptors, the ecdysone receptor (ECR) and ultraspiracle, and directly regulates target gene transcription [29–31].

ECR is a conserved gene in arthropods, and either two or three isoforms are present. In *D. melanogaster*, three nuclear hormone receptor isoforms, ECR-A, ECR-B1and ECR-B2, are encoded by the ECR gene [30, 32]. In Ixodida, AamECR cDNA encodes three isoforms [28]. AamECRA1 and AamECRA3 have a unique 5′-sequence and contain a 3218-bp 3-UTR, which diverged to produce a unique 1166-bp terminus, 3-UTR-1, or a unique 618-bp terminus, 3′-UTR-2. However, there is evidence that *Aam*ECR2 has a poly (A) sequence in the 3′-UTR region. A previous study indicated that a partial 1429-bp ECR sequence contained poly (A) in the 3′-UTR region, including a 960-bp partial open reading frame (ORF) and 398-bp 3′-UTR. In addition, the 3′-UTR sequence of AamECR2 was different from the 3′-UTR-1 and 3′-UTR-3 of AamECR. The degree of homology between the amino acid sequences of *Haa*ECR (ATA58050.1) and AamECR (AAB94567.1, AAB94566.1) was approximately 95% using Blast from NCBI. Therefore, the partial ECR sequence ECR sequence corresponding to ECR-A2 may have a biological function in *H. asiaticum*.

Previous studies have reported that many microRNAs could bind to sites located in the 3′-UTR region of target genes but only a few miRNAs could bind to the 5′-UTR or CDS of target genes [33–35]. miRNA regulates gene expression at the post-transcriptional and translational levels [36, 37]. A previous study, conducted using *Bombyx mori*, demonstrated that let-7 plays a fundamental role in molting in arthropods [16]. Our results indicate that let-7 sequences are conserved among ticks. For this reason, the complete *Aam*ECR gene sequence and the partial *Haa*ECR-A2 gene sequence were selected for analysis of the binding sites using RNAhybrid software, and our results indicated that these sequences have the same binding sites in the 3′-UTR, suggesting that the let-7 binding sites in *Haa*ECR are located in the 3′-UTR. For this reason, the 3′-UTR of *Haa*ECR was used in the dual luciferase reporter assay. However, because the bioinformatics analysis indicated that the multiple cloning site (MCS) of the pmirGLO reporter vector interacted with let-7, the sequence that did not interact with let-7 was cloned into the pmirGLO reporter vector to create the recombinant plasmid designated RP-NC. The result of the dual luciferase reporter assay showed that the let-7 binding sites are located in the 3′-UTR (Fig. 2a). The PcDNA3.1 expression vector of ECR along with the let-7 mimic was co-transfected into BHK-CDC cells to confirm the hypothesis that let-7 regulates the expression of ECR in eukaryotic cells (Fig. 2b). The results of the reporter assay using a eukaryotic expression system demonstrated that *Haa*ECR was the target of let-7 and the binding sites are located in the 3′-UTR of *Haa*ECR.

Previous studies reported that the expression of miRNAs involved in the ecdysone pathway, including let-7, mir-100 and mir-125, are upregulated by the binding of Broad-Complex with 20E in *Drosophila* [21, 36]. The expression level of let-7 was higher before molting than after molting for both larvae and nymphs whereas ECR expression was lower before molting than after molting.

There is strong evidence that 20E, as observed for other steroid hormones, regulates molting in ticks *via* a negative feedback mechanism. In normal physiological conditions, 20E binds to the heterodimer ECR-USP/RXR (ultraspiracle/retinoid X receptor) to produce ECR, E75 and Broad-Complex C (BR-C), and so on in the normal range. The signal casing by heterodimer is transmitted to the nucleus, activates the expression of downstream genes, and causes the ecdysis cascade reaction in the 20E pathway [38, 39]. However, the overexpression of let-7 causes the downregulation of ECR and affects the signal transduction pathway of ECR. For this reason, the overexpression of let-7 can downregulate the ECR gene, affecting its signal transduction pathway, and ultimately affects mortality and the period of molting in ticks. However, among the miRNAs tested in this study, miR-2a and mir-8 were downregulated and mir-451expression was not changed. A recent study reported that a target gene of miR-2a was membrane-bound trehalase and a target gene of miR-8 was phosphoacetylglucosamine. In addition, these miRNAs and their respective targets belong to the chitin biosynthesis pathway and respond to 20E signaling [40]. However, there were no studies showing that miR-451 belongs to the molting pathway and related pathways. Therefore, agomir, which increases the expression of let-7, only affects other miRNAs which is along with let-7 in one signaling pathway and does not change the miRNAs expression of other pathways.

The effect of RNA interference on the expression of let-7 and ECR was also evaluated. Both let-7 and ECR were downregulated by dsECR injection. However, the ratio of let-7 to ECR was increased (*P *< 0.05) approximately 1.37 times in the dsECR group compared with the untreated group. These results can explain the fact that ECR is regulated by let-7. In addition, phenotypic changes indicated that ECR plays a significant role in the process of molting of ticks (Fig. 6).

In this experiment, antagomir was used to knockdown let-7 expression. In spite of the fact that let-7 was downregulated and ECR was upregulated, the rate of survival was not different between the ant-let-7 group and miR-NC group (Fig. 7d). However, RNAhybrid software and Luciferase assays revealed that these five miRNAs (miR-2a, miR-2b, miR-10, miR-305, and miR-5312) can regulate the expression of ECR *in vivo* (Fig. 9). Recently, research in human diseases and cancer has proved that miRNAs-miRNAs can build a synergistic network *via* co-regulation function modules [41–43]. However, in the life-cycle of ticks, we can find molting plays a significant role in molting. As a reporter protein of 20E in the process of molting, ECR may be regulated by many co-regulatory factors. The limited miRNAs are thought to be able to control the larger set of genes through synergism, in which multiple miRNAs work synergistically to control individual genes modules. These five miRNAs may be able to make up for the function of let-7 when let-7 is abnormal. This research may provide a new view to understand the interaction between miRNAs and genes.

## Conclusions

To the best of our knowledge, this study is the first to demonstrate that let-7 plays a major role in the molting process of *Hyalomma asiaticum*. Our results suggest that let-7 can regulate the process of molting by modulating the expression of its target ECR in ticks. However, the mechanisms underlying tick development and reproduction need to be further explored.

## Abbreviations

CAAS: Chinese Academy of Agricultural Sciences
DNA: deoxyribonucleic acid
ORF: opening reading frame
miRNA: microRNA
ECR: ecdysteroid receptor
3′-UTR: 3′-untranslated region
NC: negative control
RT-qPCR: real-time quantitative PCR
RNAi: RNA interference
DLR: dual luciferase reporter assay
